# Commentary: Integrins Modulate T Cell Receptor Signaling by Constraining Actin Flow at the Immunological Synapse

**DOI:** 10.3389/fimmu.2018.02110

**Published:** 2018-09-19

**Authors:** Aviad Ben-Shmuel, Noah Joseph, Mira Barda-Saad

**Affiliations:** The Mina and Everard Goodman Faculty of Life Sciences, Bar-Ilan University, Ramat-Gan, Israel

**Keywords:** mechanotransduction, cytoskeleton, NK cells, actin retrograde flow, SHP-1

Mechanotransduction, the process by which physical forces are converted into chemical signaling, has been observed in several cell types (1–3). This mechanism enables cells to sense their environment and rapidly respond to numerous external stimuli. The process of sensing various physical cues and translating them into signaling cascades is often mediated through the cellular cytoskeleton, which serves as a global mechanosensor (4). In lymphocytes, the cytoskeleton is critical for multiple functions. Among these are migration, tissue infiltration, formation of mature and stable immunological synapses (IS), and in the case of cytotoxic T cells and natural killer (NK) cells, secretion of cytolytic granules which lead to target cell killing (5–9). During T-cell IS formation, a distinct ring-like actin architecture can be observed. This structure consists of an outer layer known as the lamellipodium characterized by rapid centripetal actin flow, a secondary layer known as the lamellum, which is defined by slower centripetal actin flow and an actin filament arc structure associated with myosin motor proteins, and an inner cell body which is F-actin depleted (10). Murugesan et al. have shown that these regions are not completely distinct, however, and actin bundles that are embedded in the lamellipodium can give rise through the action of formins to the actin arcs visible in the lamellum (11). Centripetal actin flow, also known as actin retrograde flow (ARF) has been shown to be crucial for formation of molecular clusters that participate in activating signaling cascades, and ultimately, in the termination of signaling at the cell body (12, 13).

Interestingly, there is evidence that the forces exerted by the cytoskeleton itself can influence the phosphorylation and conformation of signaling molecules, and this mechanotransduction subsequently controls protein function, and ultimately has an impact at the cellular level. For example, Babich et al. showed that perturbing F-actin dynamics decreases Phospho Lipase C Gamma 1(PLCγ−1) phosphorylation, leading to a reduction of calcium flux in T-cells (14). Additionally, Comrie et al. demonstrated that ARF can drive the affinity maturation of Lymphocyte function-associated antigen 1 (LFA-1) at the IS(15). These studies clearly demonstrate that cytoskeletal forces can potentially act as master regulators during T-lymphocyte activation.

In this compelling report, Jankowska and colleagues show that in primary T-cells, engagement of VLA-4 or LFA-1 with their ligands significantly decelerates centripetal actin flow at the IS. This study also compliments earlier reports describing inward movement of signaling microclusters (MCs) (12, 16). Furthermore, Nguyen et al. previously demonstrated that engagement of VLA-4 with cognate ligands inhibited centralization of SLP-76 MCs by retarding centripetal actin flow (17). Jankowska et al. show that the rate of centripetal actin flow is directly correlated with the magnitude of tyrosine phosphorylation responses downstream of TCR engagement. Thus, the retardation of ARF appears to directly impact T-cell signaling. Overall, under these conditions, tyrosine phosphorylation decreased, including phosphorylation of signaling molecules critical for T-cell activation, such as Zap-70 and PLCγ-1. Jankowska and colleagues emphasize that the mechanism is counterintuitive, since engagement of integrins was shown to co-stimulate activation in T-cells. They reconcile this discrepancy by indicating that most studies that focused on integrin activation in T-cells analyzed long term contacts, in which integrins play a role in maintaining cell adhesion, and not on short term signaling events. Thus, Jankowska and colleagues present an interesting model suggesting that during the initial T-cell-target cell contact, the T-cell is unresponsive to integrin-mediated signals. This allows the cell to maintain rapid ARF, which ensures signaling cluster formation, calcium flux, and integrin affinity maturation. As the IS matures, forces exerted by the cytoskeleton lead to integrin affinity maturation; in turn, engagement of integrins with their ligands slows centripetal actin flow, dampening tyrosine phosphorylation through a negative feedback loop. This represents a fascinating regulatory mechanism at the cellular level, showing that the cytoskeleton can mediate between internal signaling and integrin affinity maturation, ultimately controlling both activation and subsequent cellular inhibition.

However, several questions remain. It is not obvious how inhibiting actomyosin retrograde flow affects signaling downstream of the TCR. Specifically, is rapid ARF necessary for tyrosine phosphorylation of signaling clusters, or is there another mechanism by which ARF characterized by a slower lamellipodial velocity dampens signaling molecule function? We recently discovered a molecular mechanism that may explain some of the recent findings by Babich et al. and Jankowska et al. on the mechanistic level (18). We showed that in NK cells, actin dynamics behave similarly to those in T-cells; rapid lamellipodial ARF was observed within activating NK cell synapses (NKIS), whereas the inhibitory NKIS displayed slower centripetal actin flow. We demonstrated a selective association between the SHP-1 phosphatase and β-actin, specifically at the inhibitory synapse, where ARF was slowest. Inhibition of actin dynamics using pharmacological inhibitors such as Jasplakinolide or Cytochalasin D, or seeding cells on stiff substrates which suppress ARF, all retained SHP-1 in a catalytically inactive, closed conformation. Hence, it is clear that both association of SHP-1 with the actin machinery, and slow centripetal actin flow, are crucial for SHP-1 enzymatic activity. Importantly, abolishing actin flow results in SHP-1 inhibition, leading to an increase in tyrosine phosphorylation of its substrates including VAV-1, and PLCγ-1/2, and NK cell activation. During activating NK cell interactions, when ARF velocity is rapid, SHP-1 was not able to associate with the actin machinery, and as we demonstrated, SHP-1 remained in a closed catalytically inactive conformation, enabling tyrosine phosphorylation of VAV-1 and PLCγ-1/2.

Hence, due to the fact that SHP-1 plays an inhibitory role in T-cell signaling (19), it is possible that the effect of integrins on slowing ARF and damping tyrosine phosphorylation could be mediated through tyrosine phosphatases such as SHP-1, which as we showed, selectively associates with slow-moving actin filaments. This is also consistent with the model of Jankowska and colleagues. The initial T-cell contact induces rapid ARF, which is not permissive for association between SHP-1 and the actin machinery, thereby enabling tyrosine phosphorylation of several signaling molecules such as Zap-70 and PLCγ-1, which serve as SHP-1 substrates (20, 21). Subsequently, actin flow induces affinity maturation of LFA-1 and VLA-4, which when engaged with their ligands act as molecular clutches that slow synaptic retrograde flow. Slower retrograde flow permits association of SHP-1 with the actin machinery, inducing a catalytically active open enzymatic conformation. Active SHP-1 dephosphorylates its substrates, lowering the lymphocyte activation level thereby completing the feedback loop.

Such a model suggests the possibility of a much larger system controlled by the cytoskeleton, which precisely tunes lymphocyte signaling. It may also explain how lymphocytes such as T-cells and NK cells rapidly survey their surroundings, and process various signals in a coordinated and organized manner in a complex immune environment. It would be interesting to study whether additional signaling molecules are influenced by the forces exerted through cytoskeletal flow, and how such force transduction ultimately tunes lymphocyte function. Additionally, more work is required to understand how ARF is regulated in the early and later stages of immune synapse formation, and how it is differentially regulated in various compartments of the IS. Finally, these novel results raise the exciting possibility of manipulating immune cell cytoskeletal networks to influence cellular function.

## Author contributions

All authors listed have made substantial, direct, and intellectual contribution to the work and approved it for publication.

### Conflict of interest statement

The authors declare that the research was conducted in the absence of any commercial or financial relationships that could be construed as a potential conflict of interest.
